# The Emergence of Rare Clinical *Aspergillus* Species in Qatar: Molecular Characterization and Antifungal Susceptibility Profiles

**DOI:** 10.3389/fmicb.2019.01677

**Published:** 2019-07-30

**Authors:** Husam Salah, Michaela Lackner, Jos Houbraken, Bart Theelen, Cornelia Lass-Flörl, Teun Boekhout, Muna Almaslamani, Saad J. Taj-Aldeen

**Affiliations:** ^1^Division of Microbiology, Department of Laboratory Medicine and Pathology, Hamad Medical Corporation, Doha, Qatar; ^2^Yeast Research, Westerdijk Fungal Biodiversity Institute, Utrecht, Netherlands; ^3^Division of Hygiene and Medical Microbiology, Medical University of Innsbruck, Innsbruck, Austria; ^4^Applied and Industrial Mycology, Westerdijk Fungal Biodiversity Institute, Utrecht, Netherlands; ^5^Institute of Biodiversity and Ecosystem Dynamics (IBED), University of Amsterdam, Netherlands; ^6^Department of Medicine, Hamad Medical Corporation, Doha, Qatar

**Keywords:** aspergillosis, molecular identification, antifungal susceptibility, Qatar, Middle East

## Abstract

*Aspergillus* are ubiquitous mold species that infect immunocompetent and immunocompromised patients. The symptoms are diverse and range from allergic reactions, bronchopulmonary infection, and bronchitis, to invasive aspergillosis. The aim of this study was to characterize 70 *Aspergillus* isolates recovered from clinical specimens of patients with various clinical conditions presented at Hamad general hospital in Doha, Qatar, by using molecular methods and to determine their *in vitro* antifungal susceptibility patterns using the Clinical and Laboratory Standards Institute (CLSI) M38-A2 reference method. Fourteen *Aspergillus* species were identified by sequencing β-tubulin and calmodulin genes, including 10 rare and cryptic species not commonly recovered from human clinical specimens. *Aspergillus welwitschiae* is reported in this study for the first time in patients with fungal rhinosinusitis (*n* = 6) and one patient with a lower respiratory infection. Moreover, *Aspergillus pseudonomius* is reported in a patient with fungal rhinosinusitis which is considered as the first report ever from clinical specimens. In addition, *Aspergillus sublatus* is reported for the first time in a patient with cystic fibrosis. In general, our *Aspergillus* strains exhibited low MIC values for most of the antifungal drugs tested. One strain of *Aspergillus fumigatus* showed high MECs for echinocandins and low MICs for the rest of the drugs tested. Another strain of *A. fumigatus* exhibited high MIC for itraconazole and categorized as non-wild type. These findings require further analysis of their molecular basis of resistance. In conclusion, reliable identification of *Aspergillus* species is achieved by using molecular sequencing, especially for the emerging rare and cryptic species. They are mostly indistinguishable by conventional methods and might exhibit variable antifungal susceptibility profiles. Moreover, investigation of the antifungal susceptibility patterns is necessary for improved antifungal therapy against aspergillosis.

## Introduction

*Aspergillus* species are common environmental fungi found in soil and decaying vegetative materials. They can infect immunocompetent (Chaturvedi et al., 2017; Emiralioglu et al., 2017; Kumar et al., 2017; Saedi et al., 2017) and immunocompromised (Taccone et al., 2015) patients. Individuals with underlying diseases or immune deficiencies can develop a variety of symptoms ranging from allergies, bronchopulmonary infections, and bronchitis, to invasive aspergillosis (IA) (Ruping et al., 2008; Guinea et al., 2010; Sugui et al., 2014). IA is associated mainly with neutropenic patients suffering from hematological malignancies (Gerson et al., 1984; Abers et al., 2016). Other risk factors include hematopoietic stem cell transplant (HSCT) (Marr et al., 2002), solid organ transplant (SOT) (Patterson et al., 2000), patients receiving prolonged high doses of corticosteroids (Palmer et al., 1991; Lewis and Kontoyiannis, 2009), human immunodeficiency virus (HIV) infection with advanced acquired immune-deficiency syndrome (AIDS) (Libanore et al., 2002) and chronic granulomatous disease (CGD) (Beaute et al., 2011). IA is associated with a high mortality rate among immunocompromised patients (Baddley et al., 2010; Kontoyiannis et al., 2010; Neofytos et al., 2013; Garcia-Vidal et al., 2015). During the last two decades, species other than *A. fumigatus*, namely, *Aspergillus flavus, Aspergillus terreus, Aspergillus niger*, and other cryptic and rare species have increasingly been isolated from clinical specimens (Lass-Florl et al., 2005; Krishnan et al., 2009; Alastruey-Izquierdo et al., 2012). This epidemiological shift is attributed to the increasing number of immunocompromised patients, advances in the detection and identification of pathogenic fungi, and the selective pressure caused by extensive use of broad-spectrum antifungal drugs (Krishnan et al., 2009; Alastruey-Izquierdo et al., 2012). Voriconazole is the first line therapy recommended for the management of IA (Patterson et al., 2016; Ullmann et al., 2018). Other alternatives are liposomal amphotericin B and isavuconazole. In patients who exhibit refractory or progressive IA after the initiation of primary therapy, an additional antifungal agent may be added or a combination of antifungal agents from different classes (e.g., a triazole and an echinocandin) may be considered (Patterson et al., 2016; Ullmann et al., 2018). Posaconazole can be used as prophylaxis for patients at high risk for IA (Patterson et al., 2016; Ullmann et al., 2018). Triazole-resistant *Aspergillus*, particularly *A. fumigatus*, became a worldwide problem, with high prevalence in Europe (Alastruey-Izquierdo et al., 2013; Abdolrasouli et al., 2018; Buil et al., 2019) and recently in the United States (Berkow et al., 2018). This poses a great challenge for clinicians in patient management. Triazole resistance in *Aspergillus* has also been reported from other parts of the world, such as India (Chowdhary et al., 2015), Iran (Seyedmousavi et al., 2013; Mohammadi et al., 2016; Nabili et al., 2016), and Tanzania (Chowdhary et al., 2014). In the Middle East, apart from Iran, triazole resistance for *A. fumigatus* has also been documented in Kuwait, a neighboring Arabian gulf country, in outdoor and hospital environments (Ahmad et al., 2014) as well as from clinical samples (Ahmad et al., 2015).

The aim of the current study was to characterize 70 *Aspergillus* species isolated from a variety of clinical specimens received at the microbiology laboratory of Hamad general hospital in Doha, Qatar, with emphasis on emerging rare species identified as human pathogens, and to determine their antifungal susceptibility patterns using the Clinical and Laboratory Standards Institute (CLSI) M38-A2 reference method.

## Materials and Methods

### Patients and Specimens

Seventy *Aspergillus species* were recovered from clinical specimens of 67 patients, including immunocompromised patients (*n* = 17, 25.4%) and immunocompetent ones with other underlying diseases (*n* = 50, 74.6%) (Table 1), presented at Hamad general hospital in Doha, Qatar, between August 2003 and November 2014 with proven or probable infection or colonization by *Aspergillus* species. The patients represented 15 nationalities, including countries from Southeast Asia (*n* = 23, 23.3%) and the Middle East (*n* = 42, 62.7%), South Africa (*n* = 1, 1.5%) and the United States (*n* = 1, 1.5%). The isolates were recovered from various clinical specimens, including respiratory samples (sputum, broncho-alveolar lavage (BAL) and bronchial wash), nose and nasal sinuses, ear, wounds, pus/abscess, eye, nail, burn, pleural fluid and an unknown culture plate of clinical specimen received for identification from an external facility (Table 1).

**Table 1:** Patients demographics, clinical data, antifungal treatment, and *Aspergillus* spp. isolated.

**Specimen number**	**Gender/age**	**Origin**	**Specimen type**	**Clinical data**	**Histopathology/ CT**	**Mortality within 30 days**	**Treatment**	***Aspergillus* species**
Q1444	M/48	Qatar	Nasal swab	Nephrotic syndrome, on immunosuppressant, fungal sinusitis	+	Alive	NA^a^	*Aspergillus terreus*
Q3252	M/62	KSA	Burn	Deep left knee burn	NA	Alive	NA	*Aspergillus flavus*
Q0098	M/49	Sudan	Tissue	TB^j^, pulmonary aspergillosis, aspergilloma, X-ray+	+	Alive	ITC^b^	*Aspergillus flavus*
Q0224	F/49	KSA	BAL^g^	Endometrial adenocarcinoma	NA	Alive	AMB^c^, CAS^d^	*Aspergillus flavus*
Q4000676	F/29	India	Sinus mucosa	Nasal polyp, diabetic	+	Alive	NA	*Aspergillus welwitschiae*
Q0180	M/22	Jordan	Nasal swab	Nasal polyp	+	Alive	NA	*Aspergillus flavus*
Q1013	M/27	India	Tissue	skull lesion, sphenoid sinus extending to pteryoid fascia	NA	Died	NA	*Aspergillus flavus*
Q0878	M/23	Qatar	Tissue	Maxillary ethmoid sinus	NA	Alive	NA	*Aspergillus flavus*
Q8000006	M/17	KSA	BAL	Cystic fibrosis	NA	Alive	NA	*Aspergillus sublatus*
Q0078	M/60	Pakistan	BW^h^	Pneumonia	NA	Alive	CAS	*Aspergillus terreus*
Q0139	M/21	India	Nasal swab	Allergic rhinosinusitis	NA	Alive	NA	*Aspergillus flavus*
Q6070	M/11	Qatar	Wound	Fracture (RTA^k^)	NA	Alive	NA	*Aspergillus citrinoterreus*
Q1129	M/43	Pakistan	Debris from nose	Fungal sinusitis	NA	Alive	NA	*Aspergillus flavus*
Q0205	M/22	Nepal	BAL	Neutropenia, pancytopenia	–	Died	NA	*Aspergillus nidulans*
Q0404	F/33	Pakistan	Tissue	Fungal sinusitis	+	Alive	NA	*Aspergillus welwitschiae*
Q0782	F/35	India	Tissue	Fungal sinusitis	+	Alive	NA	*Aspergillus pseudonomius*
Q0807	M/6	Pakistan	Tissue	Chronic granulomatous disease with *Aspergillus* brain abscess	NA	Alive	VCZ^e^+AMB	*Aspergillus fumigatus*
Q6057	M/48	Egypt	Plate Culture	Unknown	NA	Alive	NA	*Aspergillus fumigatus*
Q1047	M/27	Bangladesh	Tissue	fungal sinusitis with intracranial extension	NA	Alive	VCZ+CAS	*Aspergillus fumigatus*
Q1072	M/13	Qatar	Sputum	Cystic fibrosis	NA	Alive	NA	*Aspergillus tubingensis*
Q1332	M/30	Nepal	Pus aspirate (Brain)	Fungal sinusitis	+	Alive	FL^f^, AMB, VCZ, ITC	*Aspergillus fumigatus*
Q6746	M/29	Sudan	Sphenoid sinus swab	Fungal sinusitis	+	Alive	NA	*Aspergillus terreus*
Q0012	F/61	UAE	Tissue	Nasal polyp, breast Ca	–	Alive	NA	*Aspergillus tubingensis*
Q1374	M/35	India	Tissue	Nasal polyp	NA	Alive	NA	*Aspergillus flavus*
Q0120	M/52	Sudan	Sputum	Interstitial lung disease, aspergillosis	NA	Alive	VCZ	*Aspergillus fumigatus*
Q0140	M/47	Sudan	Wound Tissue	Brain tumor	NA	Died	CAS, FL, Miconazole, VCZ	*Aspergillus nidulans*
Q1490	F/18	Qatar	Sputum	Cystic fibrosis	NA	Alive	NA	*Aspergillus fumigatus*
Q0338	M/27	India	Tissue	fungal sinusitis	NA	Alive	AMB, ITC, VCZ	*Aspergillus flavus*
Q0334	F/15	Qatar	Sputum	Cystic fibrosis	NA	Alive	NA	*Aspergillus terreus*
Q0416	F/56	Sudan	Tissue	fungal sinusitis, CT+	+	Alive	VCZ, ITC	*Aspergillus welwitschiae*
Q0521	F/74	Qatar	Tissue	RTA	NA	Alive	AMB	*Aspergillus tamarii*
Q0609	F/36	South Africa	BW	Bronchopulmonary Aspergillosis	NA	Alive	VCZ	*Aspergillus fumigatus*
Q4672	F/18	Qatar	Sputum	Cystic fibrosis	NA	Alive	NA	*Aspergillus terreus*
Q0688	M/52	Qatar	Pleural Fluid	Lung Cancer	NA	Died	VCZ	*Aspergillus fumigatus*
Q0234A	F/37	Sudan	Tissue	Fungal sinusitis	NA	Alive	VCZ, ITC	*Aspergillus welwitschiae*
Q0234B	F/37	Sudan	Tissue	Fungal sinusitis	NA	Alive	VCZ, ITC	*Aspergillus flavus*
Q0438	M/80	Tunisia	BAL	Lung cancer	NA	Died	NA	*Aspergillus terreus*
Q0490	F/26	Sudan	Tissue	Fungal sinusitis	NA	Alive	ITC	*Aspergillus welwitschiae*
Q7406	M/4	Qatar	Ear Swab	Recurrent tonsillitis, otalgia	NA	Alive	Miconazole	*Aspergillus terreus*
Q0477	M/66	Pakistan	BAL	Interstitial lung disease	NA	Alive	NA	*Aspergillus pallidofulvus*
Q6630	F/49	Pakistan	Ear Swab	ALL^l^	NA	Alive	Miconazole	*Aspergillus chevalieri*
Q1114	F/36	India	BAL	Aspergillus pneumonia	NA	Alive	AMB, CAS, VCZ	*Aspergillus welwitschiae*
Q0725	F/39	India	Tissue	Fungal sinusitis	NA	Alive	VCZ	*Aspergillus flavus*
Q4260	F/49	Sudan	Ear Swab	Hearing loss	NA	Alive	NA	*Aspergillus terreus*
Q0567	M/72	Palestine	Exit site swab	ESRD^m^	NA	Alive	NA	*Aspergillus fumigatus*
Q1177	M/78	Qatar	BW	Chest infiltrate	NA	Alive	NA	*Aspergillus fumigatus*
Q1169	F/44	Qatar	Foot tissue	Septic shock, diabetic, ESRD, bed sore	NA	Alive	NA	*Aspergillus flavus*
Q1165	F/47	UAE	BAL	Lung fibrosis	NA	Alive	VCZ	*Aspergillus flavus*
Q6596	M/63	Qatar	BAL	Polyneuropathy	NA	Died	Anidulafungin	*Aspergillus terreus*
Q1301	F/25	Qatar	Nail	Onychomycosis	NA	Alive	NA	*Aspergillus quadrilineatus*
Q6198	M/32	Jordan	Eye swab	NA	NA	Alive	NA	*Aspergillus flavus*
Q1467	F/35	Egypt	Nasal swab	Skull base meningioma	NA	Alive	NA	*Aspergillus terreus*
Q4000006	F/55	Qatar	BW	Bronchiolar asthma	NA	Alive	Miconazole	*Aspergillus terreus*
Q0518	M/29	Sri Lanka	Ear	ASOM^n^	NA	Alive	NA	*Aspergillus flavus*
Q0333	M/38	Sri Lanka	Tissue	Nasal polyp	+	Alive	NA	*Aspergillus tubingensis*
Q2266	F/35	Pakistan	Ethmoid sinus tissue	Fungal sinusitis	+	Alive	VCZ, ITC	*Aspergillus welwitschiae*
Q6811	F/8	Qatar	Ear	Otomycosis	NA	Alive	NA	*Aspergillus terreus*
Q1651	F/18	Qatar	Sputum	Cystic fibrosis	NA	Alive	NA	*Aspergillus terreus*
Q7463	M/25	India	Ear	Ear pain	NA	Alive	NA	*Aspergillus terreus*
Q7675	21/F	Qatar	Sputum	Cystic fibrosis	NA	Alive	NA	*Aspergillus fumigatus*
Q1787	M/28	Jordan	Ethmoid sinus tissue	Fungal sinusitis	+	Alive	ITC	*Aspergillus citrinoterreus*
Q2779	M/79	Qatar	Sputum	COPD^o^, lung fibrosis	NA	Alive	NA	*Aspergillus terreus*
Q0486	M/40	India	BAL	TB	NA	Alive	NA	*Aspergillus caespitosus*
Q3118	M/60	Qatar	Ear	Chronic kidney disease	NA	Alive	NA	*Aspergillus flavus*
Q0700	M/6	Qatar	BAL	Cystic fibrosis	NA	Alive	NA	*Aspergillus fumigatus*
Q3996	F/74	Syria	Ear	Sensorineural hearing loss since birth	NA	Alive	NA	*Aspergillus terreus*
Q4145	M/42	Nepal	Sputum	RTA	NA	Alive	NA	*Aspergillus terreus*
Q0861	F/39	Sudan	BAL	Chronic cough	NA	Alive	ITC	*Aspergillus flavus*
Q5260	M/57	Sudan	ETT^i^	Colitis, Pleural effusion	NA	Died	NA	*Aspergillus citrinoterreus*
Q5254	F/48	USA	Sputum	URTI^p^	NA	Alive	NA	*Aspergillus flavus*

a *Data not Available*.

b *Itraconazole*.

c *Amphotericin B*.

d *Caspofungin*.

e *Voriconazole*.

f *Fluconazole*.

g *Broncho-Alveolar Lavage*.

h *Bronchial Wash*.

i *Endotracheal Tube secretion*.

j *Tuberculosis*.

k *Road Traffic Accident*.

l *Acute Lymphoblastic Leukemia*.

m *End Stage Renal Disease*.

n *Acute Suppurative Otitis Media*.

o *Chronic Obstructive Pulmonary Disease*.

p *Upper Respiratory Tract Infection*.

*+, Positive; –, Negative; CT, Computed Tomography*.

### Isolation and Identification

*Aspergillus species* were identified by macro and microscopy according to the laboratory standard operative protocol of the microbiology laboratory at Hamad general hospital in Qatar. Specimens were cultured on Sabouraud dextrose agar (SDA; Difco Laboratories, Detroit, MI) with and without chloramphenicol. Culture plates were incubated at 26 and 37°C and were observed daily for growth up to 10 days. Direct microscopy from clinical specimens was performed using Blankophor P fluorescent stain (Bayer AG, Germany). Cultures were preserved at −70°C using cryo-tubes (Mast Diagnostics, Bootle, Merseyside, UK) until further use.

### Molecular Identification

#### DNA Extraction

Genomic DNA was extracted as described by Bolano et al. (2001), with minor modifications. In short, *Aspergillus* biomass, which was grown on oatmeal agar (OA; home-made at Westerdijk Institute) for 5 days, was bead-beaten with sterile sand, 750 μl of lysis buffer, and 750 μl of phenol-chloroform in 2 ml screw-capped tube. The mixture was centrifuged and the supernatant was transferred to 1.5 ml Eppendorf's tube with an equal amount of ice-cold 96% ethanol. One hundred microliter of 3.0 M ice-cold sodium acetate was added, mixed gently, and stored at −20°C for 30–60 min. The mixture was then centrifuged at 4°C. The DNA pellet was air-dried and re-suspended in 100 μl Tris Ethylenediaminetetraacetic acid (TE) buffer. The solution was incubated successively at 37 and 65°C both for 10 min, and stored at −20°C. The DNA quality was checked by 1.5% agarose gel electrophoresis.

#### PCR and Sequencing

For identification of the isolates, two loci were amplified, namely β-tubulin (*BenA*), and calmodulin (*CaM*). A segment of the β-tubulin gene was amplified using primers Bt2a (5′-GGTAACCAAATCGGTGCTGCTTTC-3′) and Bt2b (5′-ACCCTCAGTGTAGTGACCCTTGGC-3′) (Glass and Donaldson, 1995), and a fragment of the calmodulin gene was amplified using primers cmd5 (5′-CCGAGTACAAGGAGGCCTTC-3′) and cmd6 (5′-CCGATAGAGGTCATAACGTGG-3′) (Hong et al., 2005). The amplification of *BenA* and *CaM* loci for some of our strains resulted in poor sequence data and these strains were identified by at least one gene (*BenA* or *CaM)*. Each PCR mixture (final volume 24 μl) contained 16.45 μl water, 0.75 μl (50 mM) Magnesium chloride, 2.5 μl 10 × PCR buffer, 1.95 μl dNTP mix (1 mM), 1.25 μl dimethyl sulfoxide (DMSO), 0.5 μl of each primer (10 μM), 0.1 μl Taq polymerase (BioTaq 5 U/μL), and 1 μl of template DNA.

The PCR and sequencing reactions were performed as described previously (Visagie et al., 2014). Sequences were identified using the Basic Local Alignment Search Tool (BLAST) of The NCBI database (NCBI, 2015). A Westerdijk Institute in-house database with the latest taxonomic names and additions was also used for identification. The sequences were then deposited to the GenBank database and accession numbers are presented in Table 2.

**Table 2:** *Aspergillus* spp. isolates with Genbank accession numbers.

**Accession number**	***Aspergillus* spp**.	**Genbank accession number**	
		**Beta tubulin (BenA)**	**Calmodulin (CaM)**
Q0098	*Aspergillus flavus*	MK159746	MK039842
Q0782	*Aspergillus pseudonomius*	MK159747	MK039858
Q0521	*Aspergillus tamarii*	MK159748	MK039859
Q0477	*Aspergillus pallidofulvus*	MK159749	MK039839
Q0224	*Aspergillus flavus*	MK159750	MK039851
Q1013	*Aspergillus flavus*	MK159751	MK039852
Q1129	*Aspergillus flavus*	MK159752	MK039848
Q3118	*Aspergillus flavus*	MK159753	MK039855
Q1374	*Aspergillus flavus*	MK159754	MK039843
Q5254	*Aspergillus flavus*	MK159755	–
Q1165	*Aspergillus flavus*	MK159756	MK039853
Q6198	*Aspergillus flavus*	MK159757	MK039856
Q0234B	*Aspergillus flavus*	MK159758	MK039845
Q1169	*Aspergillus flavus*	MK159759	MK039846
Q0338	*Aspergillus flavus*	MK159760	MK039857
Q0139	*Aspergillus flavus*	MK159761	MK039847
Q0861	*Aspergillus flavus*	MK159762	MK039854
Q3252	*Aspergillus flavus*	MK159763	MK039844
Q0725	*Aspergillus flavus*	MK159764	MK039849
Q0012	*Aspergillus tubingensis*	MK159765	MK039893
Q1072	*Aspergillus tubingensis*	MK159766	MK039894
Q0333	*Aspergillus tubingensis*	MK159767	MK039895
Q0416	*Aspergillus welwitschiae*	MK159768	MK039898
Q0490	*Aspergillus welwitschiae*	MK159769	MK039901
Q1114	*Aspergillus welwitschiae*	MK159770	MK039902
Q2266	*Aspergillus welwitschiae*	MK159771	MK039899
Q4000676	*Aspergillus welwitschiae*	MK159772	MK039900
Q8000006	*Aspergillus sublatus*	MK159773	MK039904
Q1301	*Aspergillus quadrilineatus*	MK159774	MK039905
Q0140	*Aspergillus nidulans*	MK159775	MK039906
Q0486	*Aspergillus caespitosus*	–	MK039903
Q6630	*Aspergillus chevalieri*	MK159776	MK039892
Q0078	*Aspergillus terreus*	MK159777	MK039860
Q1467	*Aspergillus terreus*	MK159778	MK039875
Q2779	*Aspergillus terreus*	MK159779	MK039863
Q6596	*Aspergillus terreus*	MK159780	MK039869
Q4000006	*Aspergillus terreus*	MK159781	MK039866
Q4145	*Aspergillus terreus*	MK159782	MK039868
Q3996	*Aspergillus terreus*	MK159783	MK039864
Q6746	*Aspergillus terreus*	MK159784	MK039874
Q4260	*Aspergillus terreus*	MK159785	MK039867
Q6811	*Aspergillus terreus*	MK159786	MK039872
Q7406	*Aspergillus terreus*	MK159787	MK039876
Q4672	*Aspergillus terreus*	–	MK039873
Q1651	*Aspergillus terreus*	MK159788	MK039870
Q7463	*Aspergillus terreus*	MK159789	MK039865
Q1444	*Aspergillus terreus*	–	MK039862
Q1787	*Aspergillus citrinoterreus*	MK159790	MK039877
Q5260	*Aspergillus citrinoterreus*	MK159791	MK039878
Q0120	*Aspergillus fumigatus*	MK159792	MK039880
Q0567	*Aspergillus fumigatus*	MK159793	MK039881
Q6057	*Aspergillus fumigatus*	MK159794	MK039891
Q0609	*Aspergillus fumigatus*	MK159795	MK039882
Q1177	*Aspergillus fumigatus*	MK159796	MK039886
Q1490	*Aspergillus fumigatus*	MK159797	MK039885
Q7675	*Aspergillus fumigatus*	MK159798	MK039890
Q0700	*Aspergillus fumigatus*	MK159799	MK039889
Q1047	*Aspergillus fumigatus*	MK159800	MK039888
Q0807	*Aspergillus fumigatus*	MK159801	MK039887
Q1332	*Aspergillus fumigatus*	MK159802	MK039884
Q0688	*Aspergillus fumigatus*	MK159803	MK039883
Q0518	*Aspergillus flavus*	–	MK039840
Q0878	*Aspergillus flavus*	–	MK039841
Q0180	*Aspergillus flavus*	–	MK039850
Q0334	*Aspergillus terreus*	–	MK039861
Q0438	*Aspergillus terreus*	–	MK039871
Q6070	*Aspergillus citrinoterreus*	–	MK039879
Q0234A	*Aspergillus welwitschiae*	–	MK039896
Q0404	*Aspergillus welwitschiae*	–	MK039897
Q0205	*Aspergillus nidulans*	–	MK039907

#### Antifungal Susceptibility

*In vitro* antifungal susceptibility testing was performed according to the CLSI M38-A2 microbroth dilution method for filamentous fungi (Clinical and Laboratory Standards Institute [CLSI], 2008). The antifungal agents tested were: amphotericin B (AMB), voriconazole (VRC), itraconazole (ITC), posaconazole (PCZ) (Sigma-Aldrich, St. Louis, MO, USA), isavuconazole (ISA; Basilea Pharmaceutica, Basel, Switzerland), anidulafungin (ANID; Pfizer Pharma), and micafungin (MICA; Astellas Pharma Inc.). All antifungal drugs were tested in concentrations ranging from 0.03 to 16 μg/ml. *Pichia kudriavzevii* (*Candida krusei*) (ATCC 6258) was used as a quality control (QC) strain as indicated in CLSI M38-A2. In addition, we tested *Aspergillus fumigatus* (ATCC 46645), a reference strain from an official culture collection with known stable MIC values. The susceptibility plates were prepared and stored at −70°C until use. Results were read after 24 and 48 h of incubation at 37°C. The minimum inhibitory concentrations (MICs) for AMB and azoles were determined as the lowest concentration of the antifungal drug that prevents any discernable growth (100% inhibition) whereas the minimum effective concentrations (MECs) for echinocandins were defined as the lowest concentration of the antifungal drug that leads to rounded compact hyphal growth compared with the unchanged growth in the control well. Visual reading of the MICs/MECs was performed with the aid of an inverted mirror (Clinical and Laboratory Standards Institute [CLSI], 2008).

*Aspergillus* MICs were analyzed using the latest epidemiological cut-off values (ECVs) proposed by CLSI (Clinical and Laboratory Standards Institute [CLSI], 2018) to determine the presence of wild type (WT) and non-wild type (NWT) strains.

## Results

### Patients Groups and Aspergillosis

Seventy *Aspergillus* strains were isolated from clinical specimens obtained from 67 patients including 40 males and 27 females. The age of female and male patients ranged from 8 to 74 (media *n* = 36) and 4 to 80 (media *n* = 36.5) years old, respectively. Eight patients were under 18 years (17, 15, 13, 11, 8, 4, and 2 patients were 6 years old) and 50% (4/8) of them suffered from cystic fibrosis (Table 1).

The majority of *Aspergillus* species were isolated from respiratory specimens (*n* = 28, 40%) and nasal sinuses (*n* = 24, 34.3%) (Table 3). Two isolates (*A. welwitschiae* and *A. flavus*) were recovered from a patient with fungal rhinosinusitis. Three strains (1 *A. fumigatus* and 2 *A. terreus*) were isolated separately from sputum samples of a patient with cystic fibrosis with 4 months interval between isolations.

**Table 3:** Occurrence of *Aspergillus* spp. in clinical specimens.

	**Respiratory**	**Fungal sinusitis**	**Wound**	**Ear**	**Brain abscess**	**Nail**	**Burn**	**Eye**	**Unknown**
*A. flavus (n = 19)*	5	9	1	2			1	1	
*A. terreus (n = 17)*	9	3		5					
*A. fumigatus(n = 12)*	7	1	1		2				1
*A. welwitschiae (n = 7)*	1	6							
*A. citrinoterreus (n = 3)*	1	1	1						
*A. tubingenesis (n = 3)*	1	2							
*A. nidulans (n = 2)*	1		1						
*A. pseudonomius (n = 1)*		1							
*A. chevalieri (n = 1)*				1					
*A. tamarii (n = 1)*			1						
*A. sublatus (n = 1)*	1								
*A. quadrilineatus (n = 1)*						1			
*A. pallidofulvus (n = 1)*	1								
*A. caespitosus (n = 1)*	1								
Total (*n* = 70)	28	23	5	8	2	1	1	1	1

Fourteen patients (20.9%) presented with IA and 55 patients with non-invasive infections. The underlying conditions of these patients were immune suppression (cancer, on immunosuppressant drugs, and diabetes), chronic pulmonary disease (tuberculosis and cystic fibrosis), pneumonia, rhinosinusitis, and onychomycosis, in addition to ear, wound, skin, and eye infections. Seventeen patients were immunocompromised (24.3%) and seven patients (10.4%) died within 30 days of diagnosis irrespective of antifungal treatment. Two of the deceased patients were infected with *Aspergilus terreus*, 2 with *Aspergillus nidulans*, and 3 patients each with *Aspergillus citrinoterreus, A. flavus*, and *A. fumigatus*, respectively. Patients' demographics, clinical information and *Aspergillus* species isolated are listed in Table 1.

Fourteen *Aspergillus* species belonging to seven sections were recovered (Table 1). In addition, we detected cryptic *Aspergillus* species in 29% of our isolates (*n* = 20) which belong to 6 species complexes namely *A. welwitschiae* (*n* = 7, 10%) (section *Nigri*), *Aspergillus tubingensis* (*n* = 3, 4.3%) (section *Nigri*), *A. citrinoterreus* (*n* = 3, 4.3%) (section *Terrei*), *A. pseudonomius* (*n* = 1, 1.4%) (section *Flavi*), *Aspergillus chevalieri* (*n* = 1, 1.4%) (section *Aspergillus*)*, A. sublatus* (1.4%) (section *Nidulantes*)*, Aspergillus quadrilineatus* (1.4%) (section *Nidulantes*)*, Aspergillus pallidofulvus* (1.4%) (section *Circumdati*), *Aspergillus tamarii* (1.4%) (section *Flavi*) and *Aspergillus caespitosus* (1.4%) (section *Nidulantes*).

*A. welwitschiae* was the most isolated cryptic species (*n* = 7, 35%), followed by *A. tubingensis* and *A. citrinoterreus* (each *n* = 3, 15%) *A. terreus* was isolated from 62.5% (5/8) of the ear specimens and 47.4% (9/19) of *A. flavus* isolates were recovered from patients presented with fungal rhinosinusitis.

### Antifungal Susceptibility

All the MICs were within the required ranges for the QC and reference strains tested. Since there are no Clinical Break Points (CBPs) available for *Aspergillus* spp. by the CLSI, MIC data were analyzed and interpreted according to the ECVs indicated in the CLSI M59-ED2 (Clinical and Laboratory Standards Institute [CLSI], 2018). There are neither CPBs nor ECVs available for ANID and MICA.

One isolate of *A. fumigatus* showed a MIC of 2.0 μg/ml for ITC and was therefore categorized as NWT (ECV = 1). Another *A. fumigatus* strain exhibited high MEC values for ANID and MICA (4 and 16 μg/ml, respectively). One *A. flavus* showed an elevated MIC of 16 μg/ml for AMB and was considered to be NWT (ECV = 4). All *A. nidulans* isolated (*n* = 2) had elevated MICs of 2.0 μg/ml to AMB. Antifungal susceptibilities were not determined for *A. chevalieri* since it repeatedly failed to grow in the susceptibility test medium. The antifungal susceptibility data are presented in Table 4.

**Table 4:** *In vitro* antifungal susceptibility results of *Aspergillus* spp.

***Aspergillus* spp. isolated (*n*)**	**Antifungal**	**Range**	**GM**	**MIC/MEC (μg/ml)**									
				**0.03**	**0.06**	**0.125**	**0.25**	**0.5**	**1**	**2**	**4**	**8**	**16**
*A. flavus (19)*	ITC	0.03–0.25	0.15	1	1	11	6						
	AMB	0.125–16	0.66			2	1	6	7	2			1
	PCZ	0.03–0.25	0.06	10	4	4	1						
	VCZ	0.03–0.25	0.17	1	2	4	9	3					
	ISA	0.03–1	0.07	11	4	2		1	1				
	ANID	0.03	0.03	19									
	MICA	0.03	0.03	19									
*A. terreus (17)*	ITC	0.03–0.125	0.07	3	7	7							
	AMB	0.25–16	0.92				2	8	3	2		1	1
	PCZ	0.03–0.06	0.04	13	4								
	VCZ	0.06–0.25	0.14		4	7	5	1					
	ISA	0.03–0.25	0.04	15	1		1						
	ANID	0.03	0.03	17									
	MICA	0.03	0.03	17									
*A. fumigatus (12)*	ITC	0.03–2	0.22	1		4	5		1	1			
	AMB	0.125–2	0.53			1	4	1	5	1			
	PCZ	0.03–0.25	0.06	5	3	3	1						
	VCZ	0.06–1	0.17		2	7	1		2				
	ISA	0.03–0.25	0.09	5	1		6						
	ANID	0.03–4	0.05	11							1		
	MICA	0.03–16	0.05	11									1
*A. welwitschiae (7)*	ITC	0.25–0.5	0.37				3	4					
	AMB	0.25	0.25				7						
	PCZ	0.06	0.06		7								
	VCZ	0.25–0.5	0.30				5	2					
	ISA	0.06–025	0.11		2	4	1						
	ANID	0.03	0.03		7								
	MICA	0.03	0.03		7								
*A. tubingensis (3)*	ITC	1	1.00						3				
	AMB	0.25–0.5	0.40				1	2					
	PCZ	0.125–0.5	0.25			1	1	1					
	VCZ	0.5	0.50					3					
	ISA	0.5–2	1.00				1			2			
	ANID	0.03	0.03	3									
	MICA	0.03	0.03	3									
*A. citrinoterreus (3)*	ITC	0.03–0.6	0.04	2	1								
	AMB	1–2	1.26						2	1			
	PCZ	0.03	0.03	3									
	VCZ	0.06–0.125	0.10		1	2							
	ISA	0.03	0.03	3									
	ANID	0.03	0.03	3									
	MICA	0.03	0.03	3									
*A. nidulans (2)*	ITC	0.125				2							
	AMB	2								2			
	PCZ	0.03–0.125		1		1							
	VCZ	0.03	0.03	2									
	ISA	0.03	0.03	2									
	ANID	0.03	0.03	2									
	MICA	0.03	0.03	2									
*A. sublatus (1)*	ITC	–	–			1							
	AMB	–	–					1					
	PCZ	–	–					1					
	VCZ	–	–			1							
	ISA	–	–				1						
	ANID	–	–			1							
	MICA	–	–				1						
*A. pseudonomius (1)*	ITC	–	–				1						
	AMB	–	–						1				
	PCZ	–	–			1							
	VCZ	–	–				1						
	ISA	–	–		1								
	ANID	–	–	1									
	MICA	–	–	1									
*A. tamarii (1)*	ITC	–	–		1								
	AMB	–	–				1						
	PCZ	–	–		1								
	VCZ	–	–				1						
	ISA	–	–	1									
	ANID	–	–	1									
	MICA	–	–	1									
*A. pallidofulvus (1)*	ITC	–	–				1						
	AMB	–	–							1			
	PCZ	–	–				1						
	VCZ	–	–				1						
	ISA	–	–				1						
	ANID	–	–	1									
	MICA	–	–	1									
*A. quadrilineatus(1)*	ITC	–	–				1						
	AMB	–	–						1				
	PCZ	–	–		1								
	VCZ	–	–				1						
	ISA	–	–	1									
	ANID	–	–	1									
	MICA	–	–	1									
*A. caespitosus(1)*	ITC	–	–		1								
	AMB	–	–						1				
	PCZ	–	–	1									
	VCZ	–	–	1									
	ISA	–	–	1									
	ANID	–	–	1									
	MICA	–	–	1									

*ITC, Itraconazole; AMB, Amphotericin B; PCZ, Posaconazole; VCZ, Voriconazole; ISA, Isavuconazole; ANID, Anidulafungin; MICA, Micafungin*.

For all *Aspergillus* isolates, the range of MICs/MECs for triazoles, AMB, and echinocandins were: ITC (0.03–2.0 μg/ml), PCZ (0.03–0.5 μg/ml), VRC (0.03–1 μg/ml), ISA (0.03–2.0 μg/ml), AMB (0.125–16 μg/ml), ANID (0.03–4.0 μg/ml) and MICA (0.03–16 μg/ml). The overall geometric mean (GM), MIC_50_ and MIC_90_ are listed in Table 5.

**Table 5:** Geometric mean (GM), MIC/MEC_50/90_, range and median of *Aspergillus* MICs.

	**ITC**	**AMB**	**PCZ**	**VCZ**	**ISA**	**ANID**	**MICA**
GM	0.15	0.68	0.06	0.17	0.06	0.03	0.03
MIC/MEC_50_	0.125	0.5	0.06	0.25	0.03	0.03	0.03
MIC/MEC_90_	0.5	2	0.125	0.5	0.25	0.03	0.03
Range	0.03–2	0.125–16	0.03–0.5	0.03–1	0.03–2	0.03–4	0.03–16
Median	0.125	0.5	0.06	0.25	0.03	0.03	0.03

*ITC, Itraconazole; AMB, Amphotericin B; PCZ, Posaconazole; VRC, Voriconazole; ISA, Isavuconazole; ANID, Anidulafungin; MICA, Micafungin; GM, Geometric mean*.

## Discussion

The present study describes the molecular identification and susceptibility patterns of 70 *Aspergillus* strains isolated from clinical specimens of 67 patients from Hamad general hospital in Qatar, including adults (88%) and pediatric patients (12%). To our knowledge, this is considered as the first study exploring the molecular identification and antifungal susceptibility profiles of clinical aspergilli in this country.

Twenty isolates (29%) of cryptic *Aspergillus* spp. were recovered from patients' samples representing 10 different species that belong to six sections. Previous studies from Spain and Brazil reported a prevalence of 14.5% (Alastruey-Izquierdo et al., 2013) and 19% (Negri et al., 2014) for cryptic *Aspergillus* spp., respectively, which is lower than what we found in the current study. The majority of the cryptic species isolated in this study were members of section *Nigri* (*n* = 10/20, 50%). No cryptic species were isolated from section *Fumigati*.

*Aspergillus pseudonomius* is reported in the current study for the first time ever from clinical specimens and was isolated from a patient with fungal rhinosinusitis proven by histopathology. It exhibited low MIC values for all the antifungal drugs tested (Table 4). Echinocandins were the most active drugs (MEC = 0.03 μg/ml) and ISA was the most active triazole drug against *A. pseudonomius* with a MIC of 0.06 μg/ml.

In addition, we report the first isolation of *A. welwitschiae* from six patients with fungal rhinosinusitis, three of them were proven by histopathology and one by computed tomography (CT) as invasive infections. *A. welwitschiae* was previously isolated from patients with respiratory infections (Pinto et al., 2018) and onychomycosis (Tsang et al., 2016). Low MICs were observed for all of the antifungal agents investigated and PCZ was the most active triazole with a MIC of 0.06 μg/ml. *A. welwitschiae* was also isolated in our study from a BAL of a patient with *Aspergillus* pneumonia.

Invasive infections caused by *A. sublatus* were previously reported from patients with HSCT (de Fontbrune et al., 2014; Chrenkova et al., 2018). Here we report it for the first time from BAL specimen of an adult cystic fibrosis patient with moderate obstructive pulmonary disease. However, we were unable to discern between colonization and infection caused by *A. sublatus*. Echinocandins MICs were relatively higher for *A. sublatus* compared to the other *Aspergillus* spp. in our set, with MIC values of 0.125 and 0.25 μg/ml for ANID and MICA, respectively. Previously reported MICs of ANID and MICA for *A. sublatus* were <0.0312 μg/ml (Chrenkova et al., 2018) which is lower than our findings. ITC and voriconazole were the most active triazoles with MICs of 0.125 μg/ml for both drugs (Table 4). *A. sublatus* belongs to *Aspergillus* section *Nidulantes* and is very closely related to *A. quadrilineatus* (Hubka et al., 2016). These species are indistinguishable by sequencing *BenA* alone (Hubka et al., 2016), whereas reliable identification can be achieved by sequencing *CaM* gene (Hubka et al., 2016). In our case, we identified *A. sublatus* by sequencing both *BenA* and *CaM* genes. AMB exhibited low MIC (0.5 μg/ml) in comparison to *A. nidulans* (2.0 μg/ml) which is known to be resistant to AMB (Van't Hek et al., 1998; Kontoyiannis et al., 2002; Bowman et al., 2006). A low AMB MIC was also reported in other studies for *A. sublatus* (de Fontbrune et al., 2014; Chrenkova et al., 2018).

*Aspergillus pallidofulvus* was isolated in the present study from a BAL sample of a patient with interstitial lung disease, which is considered as the second report of this species from clinical specimens after a previous report from India where it was isolated from a BAL sample of a patient with invasive pulmonary aspergillosis (IPA) (Masih et al., 2016). The single strain isolated in the current study showed a high MIC value for AMB (2 μg/ml), which was also observed previously for this species (Masih et al., 2016). With the available data, it was not possible to recognize *A. pallidofulvus* as the cause of infection or colonization.

The recently described *A. citrinoterreus*, which belongs to section *Terrei*, was reported mainly from patients with respiratory infections, in addition to wound, abscess, nail and sinus infections (Guinea et al., 2015; Imbert et al., 2018; Vaezi et al., 2018). In a global study of 498 strains of *A. terreus* and phenotypically related species, 6 different species of section *Terrei* were identified and *A. citrinoterreus* was the second most isolated species (8.4%) (Zoran et al., 2018). In our case, among 20 strains of members of section *Terrei*, 3 strains (15%) were identified as *A. citrinoterreus*. We report the second isolation of this species from a case of fungal rhinosinusitis which was proven by histology. This patient was treated with ITC which exhibited a low *in vitro* MIC of 0.03 μg/ml and for AMB 1 μg/ml. However, there were no data available regarding the therapeutic outcome. The second isolate of *A. citrinoterreus* was from an endotracheal secretion of a patient with colitis and pleural effusion, with no other details about the underlying diseases or the immune status. Antifungal therapy information was not available for this patient who died few days after sample collection, and low MICs were observed (Table 4) for all the tested drugs including AMB MIC of 1 μg/ml. The third case of *A. citrinoterreus* was from a wound sample of a patient who had a road traffic accident. Antifungal therapy details were not available and the isolate showed low MICs *in vitro* except for AMB which showed an elevated MIC of 2 μg/ml. It was not possible to categorize the later 2 cases as infection or colonization.

*Aspergillus tamarii* is rarely encountered as a human pathogen. It was reported from few cases of cutaneous aspergillosis (Sharma et al., 2013; Kimura et al., 2018), onychomycosis (Kristensen et al., 2005), burn wound (Renner et al., 2018), keratitis (Kredics et al., 2007), respiratory (Castro et al., 2019) and sinus infections (Paludetti et al., 1992). In the current study, we report the isolation of *A. tamarii* from wound tissue of a patient who experienced a road traffic accident. This patient received AMB with unknown treatment outcome and the *in vitro* MIC of AMB was 0.25 μg/ml. We could not determine whether *Aspergillus tamarii* was the cause of infection or a colonizer.

*Aspergillus chevalieri* is one of the most common species present in indoor environments (Hubka et al., 2013). Clinically, it has been recovered from a case of cutaneous aspergillosis (Naidu and Singh, 1994), a fatal cerebral aspergillosis case (Masih et al., 2016), and respiratory, corneal and sinus infections (Siqueira et al., 2018). In our study, we isolated *A. chevalieri* from an ear swab of a patient with acute lymphoblastic leukemia (ALL). The patient received miconazole, a topical antifungal drug.

*Aspergillus tubingensis* was found to be a major fungus associated with bronchial colonization in patients with lung disease (Reynaud-Gaubert et al., 2016). Previous reports of *A. tubingensis* were from patients with cutaneous aspergillosis (Balajee et al., 2009; Pagiotti et al., 2010), otomycosis (Szigeti et al., 2012a,b), keratitis (Dóczi et al., 2009), onychomycosis (Nouripour-Sisakht et al., 2015), and osteomyelitis (Hedayati et al., 2007). We recovered *A. tubingensis* from three patients: one with unknown underlying diseases presented with fungal rhinosinusitis and was proven by histopathology. The second patient suffered from breast cancer and presented with rhinosinusitis. It was considered as either colonization or the allergic type of *Aspergillus* rhinosinusitis due to the negative histology investigation. The third patient had cystic fibrosis with unknown status of invasion or colonization. The antifungal therapy data were unavailable for those patients. In general, low antifungal MICs were exhibited for *A. tubingensis* strains except for ISA which showed high MIC of 2.0 μg/ml for the first and the second cases. No *A. niger sensu stricto* was isolated in our set of strains.

Invasive aspergillosis caused by *A. quadrilineatus*, which is closely related to *A. nidulans*, was previously reported from 2 patients who presented with fungal rhinosinusitis and had undergone bone marrow transplantation for hematological malignancy (Polacheck et al., 1992; Drakos et al., 1993), three cases of IPA in patients with CGD (Verweij et al., 2008), a patient with cerebral aspergillosis (Verweij et al., 2008) and another with onychomycosis (Gugnani et al., 2004). Our isolate was recovered from a case of onychomycosis, however, it was not possible to confirm that *A. quadrilineatus* was the direct cause of infection or colonization. A lower MIC value for AMB was observed (1 μg/ml) in comparison to *A. nidulans* (2 μg/ml) which is in agreement with a previous report (Verweij et al., 2008).

*Aspergillus caespitosus* is a soil fungus (Raper and Thoms, 1944; Chen et al., 2016) and has not been reported previously as a human pathogen. In the current study, it was isolated from a BAL specimen of a patient suffering from tuberculosis and showed low MICs for all the antifungal drugs tested. It was unknown whether *A. caespitosus* was the cause of true infection or colonization.

*Aspergillus fumigatus* has been reported as the most prevalent species causing IA in different parts of the world, including the United States, Europe and Brazil (Balajee et al., 2009; Alastruey-Izquierdo et al., 2013; Negri et al., 2014). In this study, *A. flavus* was the most prevalent species (27%), which is consistent with reports from India (47%) (Xess et al., 2004), Iran (75%) (Zanganeh et al., 2018), and Tunisia (79%) (Hadrich et al., 2010). The predominance of *A. flavus* in these parts of the world is attributed to arid and semi-arid climates (Kameswaran et al., 1992; Hedayati et al., 2007). The second most prevalent species in our set was *A. terreus* (24%), followed by *A. fumigatus* (17%). This could be due to ecological preferences for environments specific to Qatar, such as the highly arid deserts, which needs to be investigated thoroughly by an environmental sampling of different ecological niches. *A. terreus* and *A. fumigatus* were the most isolated species (29%) from immunocompromised patients and *A. terreus* was the most recovered species from respiratory samples (9/27, 33%), followed by *A. fumigatus* (6/27, 22%). Infections caused by *A. terreus* are of concern due to its reduced susceptibility to AMB *in vitro* and *in vivo* (Sutton et al., 1999; Walsh et al., 2003). This species was also found to be prevalent in Tyrol, Austria, from environmental and clinical sources (Lass-Florl et al., 2005; Lackner et al., 2016). Moreover, a previous multicentre study from the United States reported that the incidence of *A. terreus* following HSCT and SOT was found to be 16 and 11.8%, respectively (Morgan et al., 2005).

The majority of *Aspergillus* spp. were isolated from patients with non-invasive infections and a low rate of IA was observed in our study. *Aspergillus* rhinosinusitis was the second highest clinical presentation (23/67, 34.3%) after respiratory infections. These findings are in accordance with a report by Taj-Aldeen et al. (2015), which estimated the burden of fungal infections in Qatar (Taj-Aldeen et al., 2015). The study reported that *Aspergillus* rhinosinusitis in Qatar has a relatively high rate (2.31 cases/100,000 individuals). This is attributed to the hot and arid climate in the country and atopic young patients who develop allergic *Aspergillus* rhinosinusitis, in addition to the high number of residents who came from countries with elevated incidences of *Aspergillus* rhinosinusitis (Taj-Aldeen et al., 2003, 2004, 2015). The human population of Qatar is extremely mixed with high number of Southeast Asians, particularly Indians. In our set, the majority (13/23, 57%) of patients affected with *Aspergillus* rhinosinusitis originated from Southeast Asia and more than half of them (*n* = 7/13, 54%) were from India. Additionally, 4/13 (31%), patients were from Sudan. Previous reports showed that *Aspergillus* rhinosinusitis is common in these regions (Milošev et al., 1969; El Daoud et al., 1973; Chatterjee and Chakrabarti, 2010; Garg et al., 2013; Chakrabarti et al., 2015; Jain et al., 2015; Krishnan et al., 2015; Mahgoub et al., 2016). Two patients with *Aspergillus* rhinosinusitis, one of which infected with *A. flavus* and the other by *A. fumigatus*, died due to extension of the fungus to the brain (Table 1). Two patients were immunocompromised, one patient who was infected with *A. tubingensis* suffered from breast cancer. The other patient presented with nephrotic syndrome and received immunosuppressive therapy, and was infected with *A. terreus*. The latter case was proven by histology (Table 1).

Taj-Aldeen et al. (2015) also showed that among respiratory aspergillosis in Qatar the rate of infection for chronic pulmonary aspergillosis post-tuberculosis (CPA-TB) was 0.75/100 000 and the other CPA was 26.82/100,000. Allergic bronchopulmonary aspergillosis (ABPA) and severe asthma with fungal sensitization (SAFS) were more common at 60.2/100 000 and 79.46/100 000, respectively (Taj-Aldeen et al., 2015). We were unable to retrieve the complete set of clinical details about the manifestation of aspergillosis. The available clinical presentations are presented in Table 1. Overall, most of the isolates showed low MIC values for the systemic antifungal agents investigated. PCZ was the most active drug with MICs ranging from 0.03 to 0.5 μg/ml (Table 5). Echinocandins are generally potent against *Aspergillus* spp. and are used as salvage therapy or in combination with other classes of antifungal drugs (Patterson et al., 2016). However, a recent report detected a point mutation in the fks1 gene of a strain of *A. fumigatus*, which caused echinocandin resistance and subsequent treatment failure (Jimenez-Ortigosa et al., 2017). We observed echinocandin resistance in one strain of *A. fumigatus* with high MECs for ANID and MICA i.e., 4.0 and 16 μg/ml, respectively. Another strain of *A. fumigatus* showed a MIC of 2.0 μg/ml for ITC and was categorized as NWT based on CLSI ECVs (Clinical and Laboratory Standards Institute [CLSI], 2018). VRC, POS, and ISA MICs for the same strain were 1, 0.25 and 0.25 μg/ml, respectively, which were categorized as WT based on CLSI ECVs (Espinel-Ingroff et al., 2010). These findings need to be investigated by analyzing the molecular mechanism(s) of resistance. One *A. flavus* strain showed high MIC of 16 μg/ml for AMB and therefore is considered to be non-wild type (NWT) based on CLSI ECVs. Previous studies have shown that *in vitro* resistance of *A. flavus* to AMB is correlated with treatment failure (Hadrich et al., 2012; Barchiesi et al., 2013). Most of the *A. terreus* strains, a species that is considered as intrinsically resistant to AMB (Lass-Florl et al., 2005), showed high MIC values for AMB (range; 0.25–8.0 μg/ml). *A. chevalieri* failed to grow in susceptibility medium due to its xerophilic nature, therefore, no MIC values were determined.

Patients' therapeutic outcome was not calculated in our study since the data set was incomplete. We were able to retrieve the antifungal therapeutic regimes for 26 out of 67 patients indicated in Table 1. Therapy was dependent on the site of infection and underlying disease. In short, patients with IA (*n* = 6): 2 received ITC, 2 ITC, and VCZ, 1 received ITC in addition to AMB, VCZ and fluconazole, and 1 was treated with VCZ and caspofungin; immunocompromised patients (*n* = 5): 1 was treated with AMB and caspofungin, 1 with VCZ and AMB, 1 with caspofungin, fluconazole, miconazole and VCZ, and 2 patients each with VCZ and miconazole; patients diagnosed with fungal rhinosinusitis (*n* = 9): 8 received either VCZ and/or ITC, and 1 treated with VCZ in addition to caspofungin. Patients with respiratory *Aspergillus* infection or colonization (*n* = 11): 4 received VCZ, 2 treated with ITC, 1 with caspofungin, 1 with caspofungin and AMB, 1 with AMB, caspofungin and VCZ, 1 with ANID, and 1 patient received miconazole probably for a superficial infection. Among 3 patients who died within 30 days of diagnosis, 1 was treated with caspofungin, miconazole and VCZ, 1 with VCZ, and 1 with ANID. For patients treated with multiple antifungal drugs, it was unknown whether the drugs were administered singly or in combinations.

The current study highlights the molecular identification and antifungal susceptibility profiles of 70 clinical *Aspergillus* species in Qatar. Future studies with larger sample size, including clinical and environmental samples, would provide more insight into the epidemiology of clinical aspergilli in the country.

## Conclusion

In conclusion, we report the molecular identification and *in vitro* antifungal susceptibility profiles of 70 *Aspergillus* spp. recovered from various clinical specimens in Qatar. Rare and cryptic *Aspergillus* species with variable antifungal susceptibilities were detected. Triazole resistance, and recently Echinocandin resistance, is emerging in many parts of the world. Further investigation of resistance mechanism(s) is warranted for species with reduced susceptibilities to antifungal drugs. Infectious disease physicians must be aware of the emerging and resistant species to decide on accurate treatment and improved clinical outcomes.

## Data Availability

The datasets generated for this study were deposited in Genbank. The accession numbers are listed in Table 2.

## Author Contributions

HS performed the technical work and wrote the manuscript draft. JH, ML, and BT provided technical assistance. ST-A and TB assisted in designing the manuscript and writing the first draft. MA and CL-F advised on clinical aspects of the manuscript. All authors contributed to manuscript revision, editing, and approved the submitted version.

### Conflict of Interest Statement

The authors declare that the research was conducted in the absence of any commercial or financial relationships that could be construed as a potential conflict of interest.
